# Cochlea sparing effects of intensity modulated radiation therapy in head and neck cancers patients: a long-term follow-up study

**DOI:** 10.1186/s40463-014-0030-x

**Published:** 2014-08-06

**Authors:** Eleonoor AR Theunissen, Charlotte L Zuur, Marta Lopez Yurda, Sieberen van der Baan, Anne F Kornman, Jan Paul de Boer, Alfons JM Balm, Coen RN Rasch, Wouter A Dreschler

**Affiliations:** 1 Department of Head and Neck Oncology and Surgery, The Netherlands Cancer Institute, Plesmanlaan 121, Amsterdam, 1066, CX, The Netherlands; 2 Department of Maxillofacial Surgery, Academic Medical Center, University of Amsterdam, Meibergdreef 9, Amsterdam, 1105, AZ, The Netherlands; 3 Department of Epidemiology and Biostatistics, The Netherlands Cancer Institute, Plesmanlaan 121, Amsterdam, 1066, CX, The Netherlands; 4 Department of Otorhinolaryngology, Academic Medical Center, University of Amsterdam, Meibergdreef 9, Amsterdam, 1105, AZ, The Netherlands; 5 Department of Medical Oncology, The Netherlands Cancer Institute, Plesmanlaan 121, Amsterdam, 1066, CX, The Netherlands; 6 Department of Radiation Oncology, Academic Medical Center, University of Amsterdam, Meibergdreef 9, Amsterdam, 1105, AZ, The Netherlands; 7 Department of Audiology, Academic Medical Center, University of Amsterdam, Meibergdreef 9, Amsterdam, 1105, AZ, The Netherlands

**Keywords:** Radiotherapy, IMRT, Hearing loss, Cochlea

## Abstract

**Background:**

Radiation to the inner ear may lead to (irreversible) sensorineural hearing loss. The purpose of this study was to evaluate the long-term effect of radiotherapy on hearing in patients treated with Intensity Modulated Radiation Therapy (IMRT), sparing the inner ear from high radiation dose as much as possible.

**Methods:**

Between 2003 and 2006, 101 patients with head and neck cancer were treated with IMRT. Audiometry was performed before, short-term, and long-term after treatment. Data were compared to normal hearing levels according to the International Organisation for Standardization (ISO). Statistical analysis was done using repeated measurements. None of the patients received chemotherapy.

**Results:**

In 36 patients an audiogram at long-term follow-up (median 7.6 years) was available. The mean dose to the cochlea was 17.8 Gy (1.0-66.6 Gy). A hearing deterioration of 1.8 dB at Pure Tone Average (PTA) 0.5-1-2 kHz (*p* = 0.11), 2.3 dB at PTA 1-2-4 kHz (*p* = 0.02), and 4.4 dB at PTA 8-10-12.5 kHz (*p* = 0.01) was found. According to the ISO, the expected age-related hearing loss was 2.7, 4.8, and 8.8 dB at PTA 0.5-1-2 kHz, 1-2-4 kHz, and 8-10-12.5 kHz, respectively.

**Conclusions:**

After IMRT with radiation dose constraint to the cochlea, potential long-term adverse effects of IMRT remained subclinical. The progressive hearing loss over time was mild and could be attributed to the natural effects of ageing. Therefore, we recommend that a dose constraint to the cochlea should be incorporated in the head and neck radiotherapy protocols.

## Background

Radiotherapy (RT), as single-modality treatment or adjuvant to surgery, is a common treatment modality for head and neck (H&N) cancer [1]. Hearing loss is one of the adverse events of RT used in the management of H&N malignancies as the auditory system is often included in the treatment area. As a result, conductive hearing loss (CHL) may be the (reversible) effect of RT to the middle and external ear [2],[3]. In addition, radiation to the inner ear may lead to (irreversible) sensorineural hearing loss (SNHL). Recent systematic reviews reported incidences of SNHL of 42 ± 3% after RT [4],[5]. It is well known that a higher dose to the cochlea is associated with a higher risk of SNHL [1],[3],[4],[6]-[9], with a minimum cochlear dose reported to be a risk factor of 45 Gray (Gy) [4].

Currently, the use of Intensity Modulated Radiation Technique (IMRT) spares the organs at risk from high radiation doses, which can improve quality of life. Such improvements have been demonstrated in the aspect of preservation of salivary function, trismus, and neck fibrosis [10]-[12]. Equally so, IMRT will reduce the dose to the cochlea, if possible, and therefore the risk of SNHL. The advantage of IMRT on hearing status short-term after treatment is reported by different authors who compared the use of IMRT with conventional or conformal techniques in patients with H&N cancer [4],[8],[12],[13]. In a prospective study of Zuur et al., 101 patients with head and neck cancer were treated with IMRT, while sparing the inner ear from radiation dose as much as possible [6]. The radiation-induced hearing deterioration was found to be rather modest, namely 1.5 decibel (dB) at speech frequencies and 2.7 dB at ultra-high frequencies, indicating that IMRT is a safe treatment modality concerning the hearing status.

Vascular insufficiency has been proposed as the etiology of SNHL after radiotherapy. Animal studies showed that this may cause lesions in the stria vascularis, in afferent nerve endings, and in the hair cells of the cochlea [12],[14]. In long-term follow-up studies showing a progressive SNHL after conventional or conformal RT techniques, it is hypothesized that this toxicity is either caused by an increased progression of impaired circulation, or that a late onset of cochlear pathology is playing a role [2],[6],[15]-[17]. To elucidate a long-term beneficial effect of IMRT, we evaluated in the present study the same patients of our earlier published IMRT patient cohort [6], at median 7.6 years post-treatment.

## Methods

Between 2003 and 2006, 101 patients received IMRT for head and neck cancer at different tumor sites, i.e. parotid gland, oropharynx, larynx, oral cavity, maxillary sinus, submandibular gland, nasal cavity, and external ear. Audiometry was conducted in a prospective setting one week before treatment (BT), and at a median of 3.5 months (range 1.0-14.1 months) short-term (ST) post-treatment. Audiometry at long-term follow-up was defined as an audiogram at more than three years after completion of the treatment. When more than one long-term audiometry was available in one patient, the latest audiogram was used for analysis. The study was approved by the local ethics committee and an informed consent was signed by all patients before treatment.

### Intensity modulated radiation technique protocol

Computed tomography-generated treatment plans were made for all patients. The computed tomography data sets were transferred to the treatment planning systems (UM plan, version 3.38, University of Michigan, Ann Arbor, MI; and Pinnacle, version 7.3, Philips, Best, The Netherlands). The clinical target volumes (primary tumor and neck lymph nodes on both sides) and the organs at risk (parotid glands, oral cavity, brain stem, spinal cord, and the cochleae), were delineated on each relevant computed tomography slice. Thereafter, RT doses to the cochleae were calculated. For more details we refer to the previous study [6]. None of the patients received neoadjuvant or adjuvant chemotherapy.

### Audiometry

Testing was performed in a soundproof testing room with Decos system (Audiology Workstation). Both ears were tested. Air conduction (AC) thresholds were measured at frequencies 0.125, 0.250, 0.5, 1, 2, 3, 4, 6, 8, 10, 12.5 kHz and bone conduction (BC) thresholds were measured at 0.5, 1, 2, 4 kHz. Audiometric data were presented in dB Hearing Levels (HL) at frequencies 0.125 to 8 kHz and in dB Sound Pressure Levels (SPL) at frequencies 8 to 12.5 kHz. If measurements at 3 and 6 kHz were missing (72% at 3 kHz, 85% at 6 kHz) interpolation of the data was performed [11]. In case of missing measurements at high frequencies (8% at 10 kHz and 8% at 12.5 kHz) or when there was no response to the maximum output of the audiometer (4% at 8 kHz, 19% at 10 kHz, and 50% at 12.5 kHz), we calculated the thresholds by extrapolating the data, using a straight line with the same slope that was found on average in the patients who responded at all frequencies. Speech perception was not routinely measured.

Mean AC thresholds were calculated at three Pure Tone Averages (PTAs): 0.5-1-2 kHz, 1-2-4 kHz, and 8-10-12.5 kHz, chosen to estimate the expected degree of disability for speech perception in quiet, speech perception in noise, and the perception of high sounds (e.g. music, nature), respectively. We calculated mean BC thresholds at PTAs 0.5-1-2 kHz and 1-2-4 kHz. We used dB SPL values for calculating the average PTA 8-10-12.5, while we used dB HL values for the PTA of speech frequencies. An air bone gap (ABG) was calculated by the difference between AC and BC at PTA 0.5-1-2 kHz.

Audiological data were compared to normal hearing levels according to the International Organization for Standardization (ISO) standard 7029:2000 for frequencies 0.125 to 8 kHz and to a model of hearing threshold levels based on otologically unscreened, non-occupationally noise-exposed population in Sweden for frequencies 8, 10 and 12.5 kHz [18],[19]. The ISO hearing levels were calculated per patient and per frequency at baseline, short-term follow-up, and long-term follow-up.

### Otological examination

At long-term follow-up, both ears of a patient were examined with otoscopy by a head and neck surgeon. The presence or absence of the following items were scored: tympanic membrane perforation, otitis media with effusion (OME), acute otitis media (AOM), external otitis, chronic otitis media (COM), atelectasis, tympanosclerosis, stenosis of external auditory canal, and skin lesions like erythema, desquamation, eczema, and ulcerations.

### Grading of hearing impairment

Hearing impairment was expressed using the Common Terminology Criteria for Adverse Events version 4.0 (CTCAEv4) [20]. The CTCAEv4 for hearing impairment consist of four grades, based on threshold shifts at frequencies between 1 and 8 kHz: Grade 1 = threshold shift of 15–25 dB averaged at two contiguous frequencies in at least in one ear or a subjective change in hearing; Grade 2 = threshold shift of >25 dB averaged at two contiguous frequencies in at least in one ear; Grade 3 = threshold shift of >25 dB averaged at three contiguous frequencies in at least in one ear; Grade 4 = profound bilateral hearing loss (>80 dB at 2 kHz and above).

### Statistical analysis

The differences between hearing thresholds at baseline, short-term follow-up, and long-term follow-up were assessed using repeated measurement analysis. In the repeated measures analysis we adjusted for ear, gender, age and hearing level at the earliest of the measurements, and time between both measurements. A *p*-value <0.05 was considered statistically significant. Statistical analyses were performed using IBM SPSS version 20 and SAS version 9.2.

## Results

### Patient selection

In 36 of the 101 patients (36%), audiometry at long-term follow-up was obtained. Sixty-five patients (65%) were deceased, lost to follow-up, or did not want to participate anymore. Patient and treatment characteristics are shown in Table 1. Pure tone audiometry was conducted one week before treatment (BT), at a median of 3.5 months (1.0-14.1) as short-term (ST) follow-up, and at a median of 7.6 years (3.7-9.3) as long-term (LT) follow-up after treatment. Age at short-term follow-up ranged from 32–78 years, with a median age of 59 years. Patients were median 66 years old (39–85) at long-term follow-up. The mean dose to the cochlea was 17.8 Gy (1.0-66.6 Gy). Three patients received a radiation dose to the cochlea of more than 45 Gy because of the location and stage of the tumor: one patient had a tumor in the external hearing canal, one patient received post-operative IMRT for a muco-epidermoid carcinoma of the parotid gland, and the third patient received post-operative IMRT for pleomorphic adenoma of the parotid gland.

**Table 1:** Patient and treatment characteristics; N = 36 patients

Age (years), median (range)	
Short-term	59 (32–78)
Long-term	66 (39–85)
Gender	
Male	25 (70%)
Female	11 (30%)
Audiological follow-up, median	
Short-term (months)	3.5 (1.0–14.1)
Long-term (years)	7.6 (3.7–9.3)
Dose to cochlea in Gray	
Mean	17.8
Median	13.1
Range	1.0–66.6
Primary tumor site	
Parotid gland	11 (30%)
Oropharynx	9 (25%)
Larynx	8 (22%)
Oral cavity	3 (8%)
Maxillary sinus	1 (3%)
Submandibular gland	1 (3%)
Nasal cavity	1 (3%)
External ear	1 (3%)
Unknown primary	1 (3%)
T classification	
1	7 (19%)
2	18 (50%)
3	2 (5.5%)
4	2 (5.5%)
Unknown primary	1 (3%)
Not applicable*	6 (17%)
N classification	
0	22 (61%)
1	4 (11%)
2	4 (11%)
Not applicable*	6 (17%)

### Mean overall hearing loss

Hearing thresholds before, at short-term follow-up, and at long-term follow-up are summarized in Table 2. Overall, there were no significant changes at BC thresholds up to 4 kHz. At AC thresholds, hearing deteriorated with 1.8 dB, 2.9 dB, and 7.3 dB at low, high, and ultra-high frequencies, respectively, when audiometry at long-term follow-up was compared with audiometry at baseline. These differences were significant for PTA 1-2-4 (*p* = 0.03) and 8-10-12.5 kHz (*p* < 0.001). When AC thresholds at long-term follow-up were compared to AC thresholds at short-term follow-up, the following deteriorations were seen: 1.8 dB at PTA 0.5-1-2 kHz (*p* = 0.11), 2.3 dB at PTA 1-2-4 kHz (*p* = 0.02), and 4.4 dB at PTA 8-10-12.5 kHz (*p* = 0.01). According to the ISO, the age-related deterioration in hearing between audiometry at long-term and at short-term follow-up was expected up to 2.7 dB at PTA 0.5-1-2 kHz, 4.8 dB at PTA 1-2-4 kHz, and 8.8 dB at PTA 8-10-12.5 kHz.

**Table 2:** Measured hearing levels in decibel at pure tone averages

	***0.5-1-2 kHz AC mean (SD) median***	***1-2-4 kHz AC mean (SD) median***	***8-10-12.5 kHz mean (SD) median***	***0.5-1-2 kHz BC mean (SD) median***	***1-2-4 kHz BC mean (SD) median***
Number of ears	72	72	67	66	66
Before treatment	20.3 (13.3) 16.7	28.6 (16.8) 25.0	75.4 (23.6) 74.8	17.4 (12.2) 14.1	24.3 (35.1) 20.8
ST after treatment	20.3 (15.4) 15.8	29.2 (18.9) 25.0	78.3 (22.3) 79.0	16.6 (12.7) 13.3	22.9 (15.1) 20.9
LT after treatment	22.1 (15.7) 18.3	31.5 (19.0) 29.2	82.7 (23.3) 82.9	17.3 (12.9) 13.3	23.2 (15.1) 20.0
Difference ST and BT	0 dB	0.6 dB	2.9 dB*	−0.8 dB	−1.4 dB
Difference LT and ST	1.8 dB	2.3 dB*	4.4 dB*	0.7 dB	0.3 dB

*Abbreviations*: ST Short-term, LT Long-term, BT Before Treatment, AC Air Conduction, BC Bone Conduction, SD Standard deviation, dB Decibel.

*Statistically significant (*p* < 0.05).

### Air bone gap

**Figure 1:**
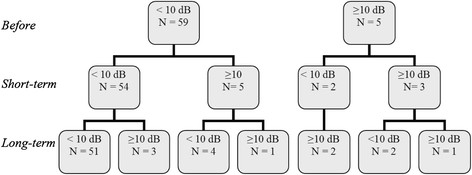
**Number of ears with an air bone gap.** Number of ears with an air bone gap at pure tone average 0.5-1-2 kHz. Measured before, short-term after treatment and long-term after treatment.

In 64/72 ears (89%) both AC and BC thresholds were measured before, at short-term follow-up, and at long-term follow-up. Before treatment, 59 ears had no ABG of which five ears developed an ABG at short-term follow-up and three ears at long-term follow-up (Figure 1). In patients with an existing ABG before therapy (n = 5), the ABG was still present (n = 3) or disappeared (n = 2) at long-term follow-up.

### Common terminology criteria for adverse events

In Table 3 hearing loss is expressed according to the CTCAEv4 for hearing impairment. Considering the audiogram LT after treatment compared to the audiogram ST after treatment, only a minor change in total incidence was seen: 39% (grade 1–3) at short-term follxow-up versus 36% (grade 1–3) at long-term follow-up. See for further details Table 3.

**Table 3:** Number of patients with hearing impairment according to the Common Terminology Criteria for Adverse Events version 4

	***Grade 0***	***Grade 1***	***Grade 2***	***Grade 3***	***Grade 4***	***Total Grade 1-4***
Short-term	22 (61%)	6 (17%)	4 (11%)	4 (11%)	0	14 (39%)
Long-term	23 (64%)	6 (17%)	4 (11%)	3 (8%)	0	13 (36%)

### Patients with progressive treatment-induced hearing loss

Two ears (3%, 2 patients) deteriorated more than expected according to the ISO at both PTAs BC, implying a treatment-induced hearing loss. At AC thresholds, three ears (5%), four ears (6%), and seven ears (10%) deteriorated more than expected according to the ISO at PTAs 0.5-1-2 kHz, 1-2-4 kHz, and 8-10-12.5 kHz, respectively (Table 4).

**Table 4:** Number of ears with treatment-related hearing loss

	***Number (ears)***	***Number (ears) with more hearing loss than expected according to ISO***	***Explanation***
PTA 0.5-1-2 kHz BC	66	2 (3%)	2x: SNHL, RT dose to cochlea >45 Gy
PTA 1-2-4 kHz BC	66	2 (3%)	2x: SNHL, RT dose to cochlea >45 Gy
PTA 0.5-1-2 kHz AC	72	3 (4%)	2x: SNHL, RT dose to cochlea >45 Gy 1x: ABG
PTA 1-2-4 kHz AC	72	4 (6%)	2x: SNHL, RT dose to cochlea >45 Gy 2x: ABG
PTA 8-10-12.5 kHz	67	7 (10%)	2x: SNHL, RT dose to cochlea >45 Gy 2x: ABG at PTA 1-2-4 kHz 3x: Unexplained

*Abbreviations*: PTA Pure Tone Average, AC Air Conduction, ISO International Organization for Standardization, SNHL Sensorineural Hearing Loss, RT Radiotherapy, ABG Air Bone Gap.

From the total cohort, three patients received a radiation dose of more than 45 Gy to the cochlea in one ear (i.e. 51.6, 52.2 and 66.6 Gy). The hearing loss of two patients was explicitly higher than the expected age-related deterioration according to the ISO: at PTA 0.5-1-2 kHz BC 11.1 dB versus 3.6 dB, at PTA 1-2-4 kHz BC 12.2 versus 6.5 dB, and at PTA 8-10-12.5 kHz 13.1 versus 6.8 dB (Table 4). The third patient lost approximately the same as expected by ISO at PTA 0.5-1-2 and 1-2-4 kHz BC. At PTA 8-10-12.5 kHz the measured hearing deterioration was 2.6 dB worse than expected according to the ISO.

### Otological adverse events

Tympanosclerosis was the most frequently observed event during otological examination (24%). An OME was seen in two patients (7%), external otitis in two patients (7%), atelectasis in three patients (10%), and skin lesions in two patients (7%). None of patients had a perforated tympanic membrane, COM, AOM, or stenosis of the external auditory canal.

## Discussion

This manuscript describes the long-term follow-up results on hearing status in a cohort of patients with head and neck cancer, treated with IMRT wherein the inner ear was defined as an organ of risk [6]. To our knowledge, this is one of the first reports describing the long-term effects after IMRT in head and neck cancer. Our results indicate that when the inner ear is regarded as an organ at risk, the treatment-induced hearing loss is modest and not progressive over time in most patients. The average change in hearing thresholds after a median interval of 7.6 years post-treatment was 1.8 - 2.3 dB at speech frequencies (PTA 0.5-1-2 kHz AC and PTA 1-2-4 kHz AC) and 4.4 dB at ultra-high frequencies (PTA 8-10-12.5 kHz) when compared to the thresholds at short-term follow-up. There were no significant changes at BC thresholds up to 4 kHz. Correction for presbycusis during follow-up time according to the ISO-standard indicated that these hearing deteriorations are part of the natural effects of ageing, as the averaged calculated hearing loss using ISO was 2.7 dB at PTA 0.5-1-2 kHz, 4.8 dB at PTA 1-2-4 kHz, and 8.8 dB at PTA 8-10-12.5 kHz. This is even higher than the measured deteriorations [18],[19].

A recent long-term follow-up study of Tsang et al. studied patients with nasopharyngeal cancer (NPC) treated with IMRT or conventional therapy on their long-term hearing status (56 ears) [21]. They concluded that there was a BC threshold shift of 16.1 dB at 4 kHz 5 years after IMRT treatment and that this deterioration, in general, could not be attributed to ageing alone. In our data, no changes were seen at BC thresholds. However, patients treated with IMRT in the Tsang study received a dose of 50 Gy to the cochlea, whereas in our study the dose to the cochlea was 17.8 Gy. This difference in radiation dose is probably related to the inclusion of only patients with NPC, whereas we included various head and neck tumor locations without any NPC patients. Nevertheless, in our IMRT population, sparing of the cochlea was not always possible depending on tumor location and stage. This happened in three patients with a tumor of the external ear or in the parotid gland. Two of them developed a progressive treatment-induced hearing loss at both AC and BC thresholds at long-term follow-up (Table 4).

In our patient cohort the cochlea was regarded as an organ at risk. However, in current practice, it is not standard to constrain the radiation dose to the cochleae. Even so, the Radiotherapy Oncology Group (RTOG) has not formulated any guidelines yet regarding dose constrains to the cochlea in H&N treatment protocols. However, a limitation of IMRT is that the dose given to tissues not considered as organs at risk can be higher compared to conventional or conformal treatment plans. A study of Hitchcock revealed this effect: patients with head and neck cancer were treated with IMRT (n = 21) or conformal RT (n = 41) [22]. As no attempt was made to limit the dose to the cochlea, patients treated with IMRT had a significantly higher dose delivered to the cochlea than those treated with a conformal treatment plan. Therefore, to better preserve hearing in most patients while using IMRT, the cochlea should be recognized and treated as an organ at risk. If possible, the radiation dose to the cochlea should be limited as much as possible, preferably lower than 45 Gy [4], although an exact safe radiation threshold is still missing in the literature. Pacholke et al. described a guideline for contouring the middle and inner ear [23]. These guidelines can be of practical help to radiation oncologists.

### Limitations of the study

This study has certain limitations. In the beginning the patient group was large (101 patients), but audiometry at long-term follow-up was only available in 36 patients (36%), since 64% was deceased, lost to follow-up, or not willing to participate any longer. However, given the fact that the hearing deteriorations were rather modest, the risk of selection bias, meaning that only patients with subjective hearing complaints continued the follow-up, is very low. Furthermore, time between short-term follow-up and long-term follow-up measurements differed between patients. However, this bias was taken into account by adjusting for time between both audiograms in the statistical analysis.

Also, a more precise conclusion may be drawn when a control group and/or a patient group treated with IMRT with high radiation doses to the cochlea, was available. Currently, due to the small sample size and the relatively large number of small radiation doses to the cochlea, a comparison between clinically relevant high and small radiation doses could only be analyzed in a descriptive manner. In our former study, reporting on the total patient cohort (n = 101), we demonstrated a dose-effect relationship between increasing radiation dose and hearing loss. Nevertheless, due to a limited number of patients receiving relatively high radiation doses (median cochlear dose was 11.4 Gy), a maximum safe cochlear dose for hearing preservation could not be calculated [6]. However, we feel that current results are sufficient enough to conclude that IMRT-induced hearing loss is rather modest at both short-term and long-term follow-up, provided that the radiation dose to the cochlea is low.

Finally, in our patient cohort, only small incidences of ABGs were found. With this limited number of ABGs no reliable conclusion can be made about the occurrence of middle ear pathology long-term after IMRT. In addition, the results of otological examination showed no abnormalities. The incidence of 24% of tympanosclerosis is, in our opinion, a normal percentage as it is correlated to ear infections in the past [15]. Of the seven patients with tympanosclerosis, five (71%) reported a medical history of recurrent ear infections before the start of IMRT. Future studies are needed to review the effect of IMRT to the middle ear and Eustachian tube function.

## Conclusion

The current follow-up study of our earlier analyzed patients with head and neck cancer treated with IMRT, resulted in a smaller sample size of the patient population and a greater diversity. Nevertheless, the importance of regarding the cochlea as an organ at risk during IMRT is well established. Based on our former ànd current results, patients suffer from modest and clinical irrelevant IMRT-induced hearing loss at both short-term ànd long-term follow-up, provided that the radiation dose to the cochlea is limited. Therefore, we recommend that a dose constraint to the cochlea should be incorporated in the head and neck radiotherapy protocols.

## Abbreviations

RT: Radiotherapy

H&N: Head and neck

CHL: Conductive hearing loss

SNHL: Sensorineural hearing loss

Gy: Gray

IMRT: Intensity modulated radiation technique

dB: Decibel

BT: Before treatment

ST: Short-term

LT: Long-term

AC: Air conduction

BC: Bone conduction

HL: Hearing level

SPL: Sound pressure level

PTA: Pure tone average

ABG: Air bone gap

ISO: International organization for standardization

OME: Otitis media with effusion

AOM: Acute otitis media

COM: Chronic otitis media

CTCAEv4: Common terminology criteria for adverse events version 4.0

NPC: Nasopaharyngeal carcinoma

RTOG: Radiotherapy oncology group

## Competing interest

The authors declare that they have no competing interests.

## Authors’ contribution

ET and CZ conceived the study, participated in the design of the study, collection of the data, interpretation of the data, analysis of the data, and drafted the manuscript. ML performed the statistical analyses, participated in the interpretation of the data, and drafted the manuscript. SB and JPdB participated in the design of the study and interpretation of the data. AK collected all the data of the study and participated in the interpretation of the data. AB participated in the design of the study, interpretation of the data, and drafted the manuscript. CR and WD coordinated the study, participated in the design of the study, interpretation of the data, and drafted the manuscript. All authors read and approved the final manuscript.
